# The Effect of Cobalt on the Deformation Behaviour of a Porous TiNi-Based Alloy Obtained by Sintering

**DOI:** 10.3390/ma14247584

**Published:** 2021-12-10

**Authors:** Nadezhda Artyukhova, Sergey Anikeev, Vladimir Promakhov, Maxim Korobenkov

**Affiliations:** 1 International Research Center “Coherent X-ray Optics for Megascience Facilities”, Immanuel Kant Baltic Federal University, Alexander Nevsky Str., 14, 236016 Kaliningrad, Russia; artyukhova_nad@mail.ru (N.A.); korobenkovmv@gmail.com (M.K.); 2 Scientific and Educational Center “Additive Technologies”, National Research Tomsk State University, Lenin Avenue, 36, 634050 Tomsk, Russia; anikeev_sergey@mail.ru

**Keywords:** TiNiCo, porous material, sintering, deformation behaviour, martensitic shear, precipitation hardening

## Abstract

This research investigates the effect of cobalt on the deformation behaviour of a porous TiNi-based alloy that was obtained by sintering. Porous TiNi-based alloys with cobalt additives, accounting for 0–2 at. % and with a pitch of 0.5, were obtained. The structural-phase state of the porous material was researched by X-ray structural analysis. The effect of different amounts of Co (used as an alloying additive) on the deformation behaviour was investigated by tensile to fracture. The fractograms of fracture of the experimental samples were analysed using scanning electron microscopy. For the first time, the present research shows a diagram of the deformation of a porous TiNi-based alloy that was obtained by sintering under tensile. The stages of deformation were described according to the physical nature of the processes taking place. The effect of the cobalt-alloying additive on the change in the critical stress of martensitic shear was investigated. It was found that the behaviour of the concentration dependency of stress at concentrations under 1.5 at. % Co was determined by an increase in the stress in the TiNi solid solution. This phenomenon is attributed to the arrangement of Co atoms on the Ti sublattice, as well as an increase in the fraction of the B19′ phase in the matrix. The steep rise of the developed forces on the concentration dependency of the martensitic shear stress at 2 at. % Co is presumably attributed to the precipitation hardening of austenite due to the precipitation of finely dispersed coherent Ti_3_Ni_4_ phase following the decrease of fraction of martensite. An analysis of fractograms showed that as more cobalt was added, areas of fracture with traces of martensite plates of the B19′ phase started to prevail. At 2 at. % Co these plates fill almost the entire area of the fracture. The research findings presented in this work are of great importance, since they can be used to achieve the set of physical and mechanical properties required for the development of biocompatible materials for implantology.

## 1. Introduction

Alloys based on titanium nickelide (TiNi) have an optimal set of structural and functional properties, which allows them to be used for solving complex problems in medicine and technology [1,2,3,4,5,6]. Changing the Ni/Ti ratio and adding a third element as an alloying additive creates more opportunities for adjusting the structural characteristics of martensitic transformations, shape memory parameters and the mechanical properties of TiNi. Cobalt is an efficient means for the adjustment of these properties. Estimations of the effect of cobalt on the shape memory parameters and mechanical properties of monolithic materials, based on TiNi for medical purposes, were published in the literature [7,8,9,10]. Phukaoluan et al. showed that the addition of 3% Co in a TiNi alloy can increase the loading–unloading force of TiNi wire due to the increase in phase fraction austenite. These findings are consistent with Kok et al., who found that the martensite phase can have a cobalt ratio of up to 3 at.% in the TiNi alloy.

Porous materials based on TiNi are successfully used in medical practice along with monolithic materials. A well-developed volumetric structure of these materials plays a certain role here, since it is close in anatomical parameters to the biological bone tissues of the human body [4,11,12,13]. Changes in the parameters of shape memory effects and temperature ranges of martensitic transformations for sintered porous alloys with Co additives are covered in [14,15,16,17]. In these studies, porous-sintered TiNi-based alloys were investigated. These alloys were obtained by reaction and diffusion sintering, and the effect of Co doping on the parameters of shape modification under load, as well as the sequence and temperature range of martensitic transformations, was considered. Based on the analysis of the experimentally obtained temperature dependences of electrical resistance and the multiple (two-way) shape memory effect, a conclusion was drawn regarding the influence of cobalt on martensitic transformations in sintered alloys. It was found that the addition of cobalt transformed the martensitic transformation so that it occurred through the R-phase and consisted of two stages: B2 → R → B19. The addition of 1–2 at. % Co expands the temperature range of the martensitic transformation, shifts the temperature of the end of the martensitic transition (Mf) to the low-temperature region (as low as 77 K). Concerning the deformation under load, 0.5–1 at. % of Co increases the maximal accumulated Co and decreases the reversible strain. All of the values of the deformation modes decrease at 1–2 at. % of cobalt. It was suggested that such deformation behaviour may be associated with internal stresses in TiNi due to the addition of Co. Therefore, it is very important to study the effect of cobalt on the level of martensitic shear stress, which is directly related to internal stresses in the matrix phase. This effect is disclosed in the present study. However, the effect of Co on the mechanical properties in these materials has not been considered.

An important parameter that allows medical alloys to match the biomechanical behaviour of biological tissues is the critical martensitic shear stress. This is the stress at which martensitic transformation is initiated. Accordingly, shape memory and superelasticity effects are based on this transformation. Thus, it is of great importance to study the effect of the introduced Co on this characteristic of the material obtained.

The generally accepted method used for investigating the deformation behaviour and assessing fracture via deformation diagrams for porous materials is the compression method [17,18]. In this research, the analysis of the stages of deformation and the dynamics of the fracture process for porous sintered alloys, to the best of our knowledge, was carried out under the conditions of tensile deformation for the first time.

Thus, the purpose of this work was to study the effect of cobalt on the deformation behaviour and the critical stress of martensitic shear of a porous TiNi-based alloy obtained by sintering.

The results obtained will make it possible to fill the gap in the research data on the effect of cobalt additives on the properties of the resulting porous TiNi alloys.

## 2. Materials and Methods

**Figure 1:**
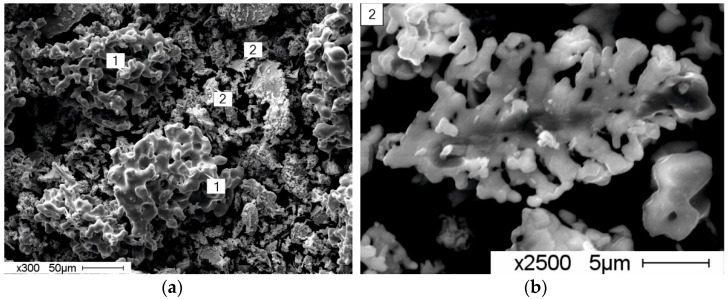
Micrographs of a mixture of powders TiNi-Co (**a**) and particle of powder Co (**b**) for sintering: (1) spongy particles TiNi; (2) particle Co is dendrite-shaped.

Samples with and without the addition of Co (0.5; 1; 1.5; 2 at. %), were obtained by diffusion sintering. To create porous alloys, we used TiNi powder of PN55T45S grade and cobalt powder of PK-IU grade. Micrographs of a mixture of powders TiNi-Co for sintering were shown in Figure 1, where the range of particle sizes of TiNi and cobalt powders was (100–140) μm and (70–100) μm, respectively. Co was used in the form of a crystalline powder intended for the production of the artifacts by powder metallurgy. It is important to note that TiNi powder is made of spongy particles (1), whereas cobalt powder particles are dendrite-shaped (2).

The amount of the alloying additive was calculated by replacing nickel (Ti_50_Ni_50−x_Co_x_); the powders were dosed using an A&D GH-200 (A&D, Tokyo, Japan) analytical balance, accuracy class I. The components of the powder mixture were pre-dried in a laboratory vacuum oven at a temperature of 60–70 °C and a pressure of 10^−4^ Pa over 4–8 h. The initial mixture was prepared with standard with V–mixers operating over 6–8 h, after which the powder was poured into sintering moulds.

The porous TiNi-based materials, were prepared using quartz tubes (12 mm in diameter) that were laid horizontally in the furnace on a graphite mould. The tubes were sealed with plugs made of porous TiNi, obtained by self-propagating, high-temperature synthesis (SHS). The initial porosity of the TiNi powder batch before sintering was 65–70% to match the porosity of the sintered material with cancellous bone tissue. The porosity, *φ*, is defined as:

(1) $$\varphi =\left(1-\frac{\rho_{porous}}{\rho_{bulk}}\right)\times 100\%$$

where ρ*_porous_* is a porous sample density, which determines by dividing the mass by its volume and  ρ*_bulk_* = 6.45 g/cm^3^. Sintering was carried out at a pressure of 6.65 × 10^−4^ Pa with an average heating rate of 10 °C/min. Diffusion sintering of TiNi powder with and without cobalt additives was carried out at a temperature of 1270 °C and a sintering time of 15 min.

To carry out mechanical tests, plates with a size of 50 × 5 × 3 mm^3^ were cut out from the obtained porous samples by electrical discharge cutting on an ARTA 153 unit (Delta Test, Russia). Fracture tests of porous sintered samples with and without the addition of cobalt were carried out on an Instron 3369 (Instron, Buckinghamshire, UK) testing machine at a strain rate of 0.1 mm/min by tensile at room temperature. The porous sample was placed in a vertical position and fixed with clamps, so that the axis of the sample was matched with the axis of the clamps. This was conducted in order to rule out warping and to ensure uniform tensile of the sample under deformation.

To determine the reliability of the results in the experiment, 9 samples of each concentration were used. It is extremely difficult to determine the absolute value of the ultimate strength of porous alloys, since porous samples are inhomogeneous in their phase composition, concentration composition, and macrostructural parameters.

Phase compositions were defined at Belgorod State Technological University (BSTU) by a Shimadzu XRD 6000 (Shimadzu, Kyoto, Japan) diffractometer using Cu Kα radiation in the range of reflection angles (2θ°) 20–100 with 0.02° scanning pitch and 1 s exposure.

Diffraction patterns were decoded, and phases were identified using the PDF-2 database of the International Center for Diffraction Data (ICDD) using the Crystallographica Search-Match (CSM) software by Oxford Cryosystems. Quantitative X-ray Diffraction (XRD) analysis was based on the use of full-profile calculation procedures implemented in the Powder Cell 2.4 software. The calculations were carried out using a standard strategy for refining instrumental and structural parameters (scale factors of phases, position of the zero point of the goniometer, refinement of the background line, parameters of the elementary cells of mineral components, profile parameters, etc.).

The fractograms of the fracture of the metal matrix of the experimental samples were investigated by scanning electron microscopy using Quanta 200 3D (Hillsboro, OR, USA) microscopes in the secondary electron mode at a voltage of 20–30 kV. The concentration composition of the phases was determined using an EDAX ECON IV energy dispersive spectrometer (EDS).

## 3. Results and Discussion

### 3.1. Structural-Phase Composition of Porous, Sintered, TiNi-Based Alloys with Co Additives

The optimal structural characteristics of the sintered TiNi-based material were obtained using a certain temperature–time mode. By focusing on the amount of the resulting liquid phase while achieving minimal shrinkage, surface melting, a high quality of interparticle contacts, and regular porosity were found empirically. In choosing the sintering temperature, the melting point of the TiNi phase was used as a reference point. According to various data from the phase diagram of the Ti-Ni system, this lay within the range of 1250 to 1310 °C. Thus, the described experimental approach made it possible to select the sintering temperature (T = 1270 °C) and time (t = 15 min) necessary to obtain the required macrostructural parameters of the sintered TiNi-based material.

**Figure 2:**
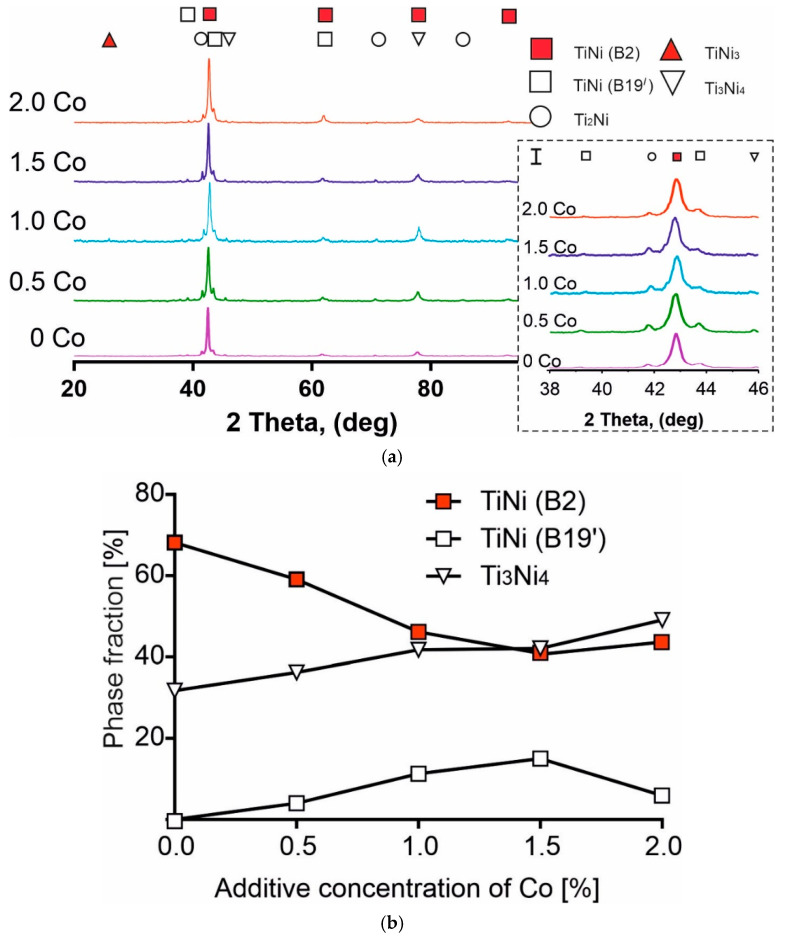
Structure-phase composition of porous TiNi-based alloys with an alloying addition of Co obtained by sintering: (**a**) X-ray diffraction patterns of the alloys with Co fractions in the range of 0–2 at. %. In the inset I (on the right) an enlarged fragment of the diffractogram in the range of 2 Theta (38–46) deg; (**b**) concentration dependency of the fraction of phases in the structure of porous alloys.

As shown by X-ray structural analysis, the structural phase composition of the investigated sintered porous alloys is represented by a single set of phases: the austenite TiNi (B2) phase; TiNi (B19′) martensitic phase; and Ti_3_Ni_4_, TiNi_3_, Ti_2_Ni phases (Figure 2a), the proportion of which varies depending on the concentration of the Co alloying additive. Figure 2b shows the concentration dependency of the fraction of the B2, B19′, and Ti_3_Ni_4_ phases in the structure of the porous alloys being researched. Only these phases from the Ti-Ni system were sampled because of their significant influence on the deformation behaviour of TiNi-based alloys. By analysing Figure 2b, it can be inferred that, for the austenite and martensite phases of the TiNi matrix phase, the critical concentration of Co is 1.5 at. %. Below this value, as more Co is added, the fraction of the austenite TiNi (B2) phase decreases while the fraction of the martensitic TiNi (B19′) phase increases. Above the critical value, the behaviour of these parameters is reversed. Figure 2b also shows an increase in the fraction of the finely dispersed lenticular phase of Ti_3_Ni_4_ with an increase in the alloying additive. The graphs change into a plateau at 1.5 at. % Co.

For all the samples with different concentrations of the alloying additive, no peaks of metallic Co appeared at X-ray diffractograms. This behaviour allows for the full completion of the reaction of Co with the TiNi and Ti2Ni components of the powder. This reaction can occur in a solid state as a diffusion, as well as with the help of the fusible Ti2Ni component.

Thus, X-ray diffraction analysis showed the following outcomes. Firstly, none of the obtained materials contained a pure cobalt phase. Therefore, all of the introduced cobalt interacted with the main component of the initial mixture—intermetallic TiNi powder. Secondly, the content of both the main matrix and secondary phases changes with an increase in the amount of the additive and passes through an extremum (most often a maximum) in the range of 1–1.5 at. % Co.

### 3.2. Schematic Representation of the Deformation Processes of Sintered Porous TiNi-Based Alloys, Breakup by Stages

The theoretical analysis of the modern understanding of the deformation of shape memory alloys carried out in this work allowed us to compare the physical nature of the processes occurring in the structure of the material with individual stages of deformation on the experimental curves. For the convenience of describing and analyzing the experimental fracture diagrams of porous TiNi-based alloys, obtained by sintering with Co additives, we introduced a generalized curve σ (ε), consisting of a basic combination of elements.

**Figure 3:**
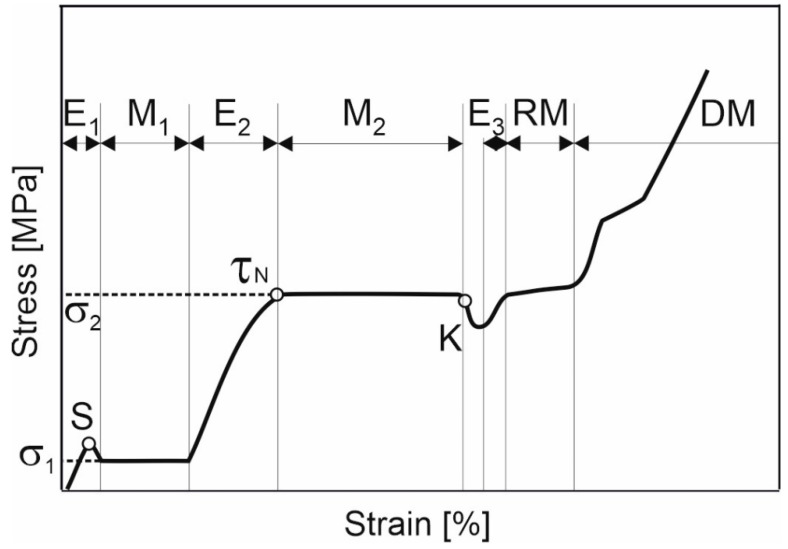
Schematic representation of a generalised stress–strain curve of a porous, TiNi-based alloy under tensile. The image consists of a basic combination of elements.

**Figure 4:**
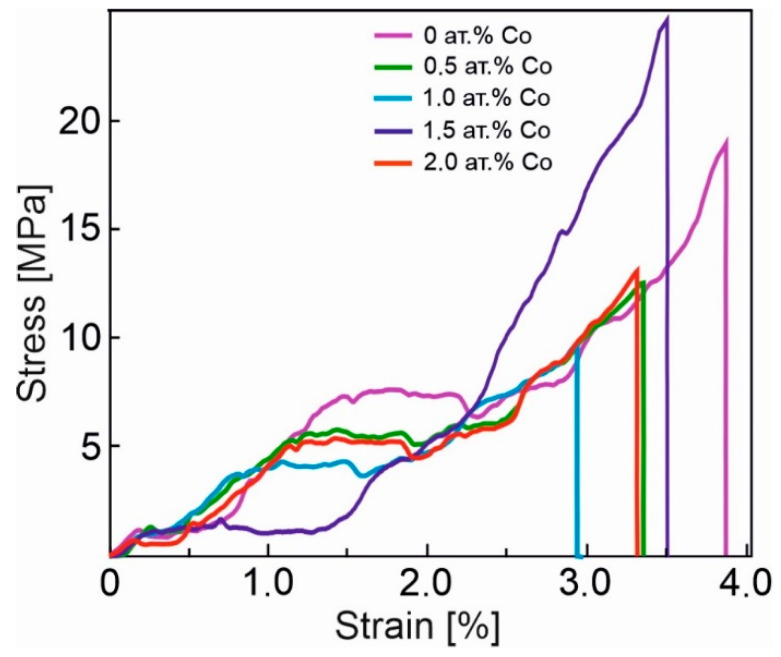
Dependencies σ(ε) of sintered, porous, TiNi-based alloys with the addition of (0–2) at. % Co under tensile.

Figure 3 is a schematic representation built on the basis of repeated stages corresponding to the experimental dependences σ (ε) in Figure 4. Each stage has a different deformation mechanism:

– Ei—areas of elastic deformation (where I ∈ Z);
– M_j_—areas of sample yield (where j ∈ Z) associated with the occurrence of martensitic transformation;
– RM—the martensite reorientation stage;
– S, τ_N_—spike and yield point;
– K—the point of stress decay after the martensitic transformation;
– RM—nonlinear deformation of reoriented martensite;
– DM—the stage involving mechanisms of plastic deformation of reoriented martensite.

The shape of the first section E_1_ in this diagram (Figure 3) is due to the elastic deformation of the initial austenitic phase. In Figure 3 it can be seen that, as the stress increases, the stage of elastic deformation is replaced by the M_1_ plateau. Stage M_1_ is consistent with the plateau of material yield upon elongation of the sample without increasing the applied forces associated with the martensitic transition during loading. The end of the plateau corresponds to the completion of the transformation. The further increase in the stress level causes the elastic deformation of the phase that has formed.

Sections Ei of the elastic deformation of the material have a certain angle of inclination that characterizes the elastic modulus E of a certain phase, whose deformation prevails in the tensile interval in question. In our opinion, the presence of two yield plateaus, M_1_ and M_2_, is associated with a two-stage martensitic transformation. Moreover, each of the plateaus has a different origin. The first plateau is associated with the development of the A → R martensitic transformation and the appearance of the R-phase in the material. The second plateau, M_2_, is related to the development of the R → M transformation and the transition of a larger volume of the porous material to the martensite phase. This interpretation of the presence of the second plateau on the deformation curve is confirmed in previous research [1,19,20,21,22,23,24]. Additionally, Pushin et al. [25] noted that monolithic ternary alloys Ti_50_Ni_50−x_Co_x_ are characterised by the emergence of an intermediate rhombohedral R-phase during the martensitic transformation: B2 → R → B19′.

An analysis of a group of experimental fracture curves for porous TiNi alloys with Co additives (Figure 4) allowed for the identification of a rather long plateau, associated with the transition to the R-phase (which is reflected in the diagram; see Figure 3). By contrast, this stage is shorter for monolithic alloys based on TiNi, since the A → R transformation is attributed to transitions that are close to type II [1,2,20,26,27]. Additionally, it is emphasised in [2,28] that the transition to the R-phase is characterised by a very narrow hysteresis (2−5 K) and small shear deformation (about 1.5%). At the same time, for a porous TiNi-based alloy, the overlapping of the following processes is possible: those associated with the appearance of the R-phase and those associated with the formation of martensite nuclei. This is due to their structural-phase inhomogeneity and inhomogeneity of loading during tensile, due to the porous structure of the material [1]. This, in our opinion, leads to the lengthening of the M_1_ plateau.

In Figure 3, it can be seen that a characteristic feature of σ(ε) dependence is the breakpoint S at the transition of the elastic section E_1_ to the M_1_ plateau. For alloys with thermoelastic martensitic transformation, the decrease in stress after the elasticity section upon reaching the peak value is associated with the instability of the austenite phase and the formation of martensite crystals in the structure [1,29,30]. Thus, stress relaxation in the sample is provided due to the formation of a martensitic phase (martensitic shear), which leads to a softening of the material and its deformation at a reduced stress. This stress value is maintained until the martensitic transformation is completed within a certain volume. In [31,32], another explanation for the stress reduction at the beginning of the martensite plateau is presented. This version is associated with the difference between the stress of martensite nucleation and the stress required for the movement of the phase transformation front during snowballing formation of martensite crystals [33]. In some cases involving the deformation of a twinned martensitic TiNi polycrystal, this behaviour of the stress characteristic is the beginning of the martensite twinning process (nucleation). Here, the deformation plateau is associated with a continuous process of twinning over the test sample (i.e., propagation). In other cases, in polycrystalline TiNi-based alloys with shape memory, the stress drop represented by the yield spike indicates the initiation (as a sharp wedge on the graph) and subsequent propagation of Luders bands, i.e., deformation bands on a macroscopic scale [34,35]. Thus, the plateau of the deformation curve in this case can be associated with a Luders-like deformation [36], but only under tensile.

In [37], section S is referred to as the yield spike, which has an upper and lower limit (see Figure 2 of [37]). Based on the theory of dislocations [38], the appearance of a yield spike is determined by the fact that at the upper yield point, and while exposed to loading, dislocations in the material begin to move along the interphase surface. The movement of the dislocations occurs via the sliding mechanism [39]. The translation of partial dislocations is associated with the rearrangement of the lattice during the movement of the interphase boundary in the course of martensitic transformation. Since the slippage of dislocations results in a lower resistance in the material, the dependency σ(ε) shows a decrease in the stress at the end of the elastic region from the upper to the lower yield point. After some time, the growing number of dislocations and defects in the material reaches the point where they restrict the movement of each other. This leads to the strain hardening of the martensite phase in this yield region [40,41].

Figure 3 shows that the section of plastic flow M_1_ is replaced by the elastic region E_2_, and the porous sample re-acquires the ability to withstand elongation. This is due to the fact that the R-phase and the strain-hardened martensite phase formed at the M_1_ stage are elastically deformed. Additionally, at this stage, residual austenite, Ti_2_Ni-based phases, and non-metallic phases can act as the “rigid” phases within the structure, and they have a brittle or quasi-brittle fracture type.

At the transition of elastic section E_2_ to the rectilinear section M_2_, the S spike degenerates into the yield point—τ_N_ (Figure 3). In our opinion, the second deformation plateau M_2_ is associated with the transition from the R-phase to martensite (stress-induced martensite). At the end of the M_2_ plateau, the transition stops and the elastic deformation of martensite starts. However, by the end of this phase transition, similar to the stress decrease in section S, there is a decrease in stress K. According to Karaca et al. [31], this decrease may be associated either with material softening as a result of the martensitic transformation R→M, or with the beginning of the reorientation of martensite in the structure of the material and the nucleation of favourably oriented martensite crystals [42,43,44]. This stress decay period is followed by a rise in the fracture curve caused by the elastic deformation of the martensite phase.

Let us consider the subsequent stages of the development of processes in the structure during the tensile of the porous sample. Areas of reorientation of martensite (RM) and the nonlinear deformation of reoriented martensite (DM) can be clearly distinguished (see Figure 3). It should be noted that the analysis of the final stage of deformation of the experimental fracture curves for porous TiNi with Co additives, as shown in Figure 4, demonstrates the absence of a clearly defined stage of plastic deformation in the material. In addition, along with the sections of pronounced deformation plates corresponding to the A → R and R → M transitions of a larger volume of material, these σ(ε) dependences have gently sloping sections displaying the full transformation of residual austenite in the porous Ti-Ni system [45]. Their appearance is caused by the inhomogeneity of the structure. This is further aggravated by the inhomogeneity of the loading of porous samples, due to the different sizes of interpore bridges in a wide range of values from 30 to 400 μm.

Thus, it was shown that the pattern of the deformation processes of a porous TiNi-based alloy under tensile is multi-stage. Accordingly, each stage has its own characteristics of deformational behaviour that are in line with the physical nature of the processes occurring during the course of the stage. The main stages of the scheme are sections of elastic deformation, deformation plateaus corresponding to the transitions A → R and R → M areas of reorientation of martensite, as well as stress decreases before and after the yield plateaus. The peculiarities in the behaviour of the experimental curves at the final stage of deformation were determined. Firstly, this is the absence of the plastic deformation stage caused by a large number of phases with brittle and quasi-brittle types of fracture (residual austenite, secondary and non-metallic phases). Secondly, there are gently sloping areas of residual austenite transformation due to structural inhomogeneities and loading in porous alloys.

### 3.3. Concentration Dependency of the Martensitic Shear Stress in Porous Sintered TiNi-Based Alloys with Co Additives

In this research, it is shown that the addition of cobalt changes the behaviour of the fracture diagram for a porous, TiNi-based alloy, obtained by sintering (Figure 4). One of the factors affecting the type of dependency σ(ε) is the level of stresses corresponding to the martensitic transition (M_1_ and M_2_).

It is clear that the dependence of the stresses σ_1_ associated with the first yield plateau M_1_ (A → R) (AR) on the addition of cobalt is weak. By contrast, the stress that is characteristic of the second plateau M_2_–σ_2_ (the stress of formation of martensite R → M) changes significantly with the addition of cobalt. This stress (σ_2_) can be considered as the critical stress of martensitic shear, and the deformation associated with this stress can be considered as martensitic deformation.

**Figure 5:**
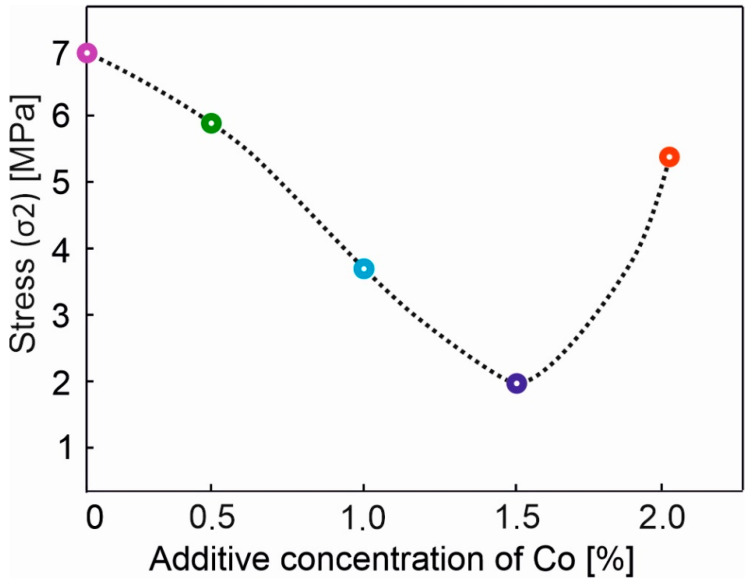
Concentration dependency of stress σ_2_ corresponding to the martensitic transition R → M.

Based on the fracture diagrams for porous, sintered alloys, which are TiNi-based with Co additives, the concentration dependency of the stress σ_2_ corresponding to the martensitic transition R→M was plotted (Figure 5). Analysis of this dependence distinguishes two peculiarities associated with the concentration points of 0.5 at. % and 2 at. % Co. With the addition of 0.5 at. % Co, a sharp decrease in the stress σ_2_ is observed. The downward trend in the stress is maintained up to 1.5 at. % Co. With the addition of 2 at. % Co, its numerical value rises to the previous level, corresponding to 0.5 at. % Co. This behaviour of the stress value at which martensite is formed (σ_2_ at these concentrations) correlates with the change in the values of the electrical resistivity, as well as the accumulated, residual and reversible deformations of shape memory materials for sintered alloys with cobalt additives [15].

A concentration behaviour of up to 1.5 at. % Co (Figure 5) is presumably associated with an increase in the stress in the TiNi solid solution. This can be explained by the predominant position of Co atoms at the Ti sublattice [1,46] and a consequent stress in the matrix. An increased stress in austenite leads to a reduction in minimal martensitic shear stress and promotes the martensitic transformation within the sample at moderate forces. This can be attributed to the formation of intermetallic compounds with titanium, while only solid solutions are formed with Ni [46]. In addition, the change in the material properties is directly related to the peculiarities of the microstructure of sintered porous alloys with Co additives. More specifically, the stress required for the formation of stress-induced martensite depends on the changes in the fractions of such phases as TiNi (B2), TiNi (B19′), and Ti_3_Ni_4_. Thus, according to the authors, a decrease in martensitic shear stress is related to an increase in the proportion of the martensitic TiNi (B19′) phase and a decrease in the proportion of austenitic TiNi (B2) phase (see Figure 2). This favors the elasticity of the material with 1.5 at. % doping. One may observe, in Figure 4, for this concentration of cobalt, that the decrease in the developed forces is represented by the merge of the first (σ_1_) and second (σ_2_) plateau in the yield diagram σ(ε) of the porous alloy (the violet line). The steep increase in the developed forces on the concentration dependency of the martensitic shear stress at 2 at. % Co is presumably attributed to the precipitation hardening of austenite due to the precipitation of the finely dispersed coherent Ti_3_Ni_4_ phase, following the decrease in the fraction of martensite (see Figure 2b). Clearly, this leads to a decrease in the stress in the TiNi solid solution and its return to the level characteristic of the minimally alloyed 0.5 at. % alloy.

Thus, from the above analysis, two critical concentrations of cobalt were determined, and they are 0.5 and 2 at. %. It was shown that the addition of 0.5 at. % Co initiates the process of the reduction in the critical stress of martensitic shear. Meanwhile, the addition of 2 at. % Co triggers the return to the previous value corresponding to 0.5 at. % Co. It was found that the behaviour of the concentration dependency of σ_2_ is influenced by two main processes: an increase in the proportion of martensite in the alloy matrix and the precipitation hardening of austenite. For values up to 1.5 at. % of Co the first process predominates. Above that, the hardening of austenite by the precipitation hardening mechanism has an effect. From the analysis that was carried out during the present study, the addition of 1.5 at. % of cobalt was highlighted as the value at which the critical stress of martensitic shear is reduced. This, in turn, increased the elasticity of the porous TiNi alloy.

### 3.4. Fractograms of the Fracture Surface of Porous TiNi-Based Alloys with Co Additives

The specific features of the tensile fracture diagrams of porous sintered TiNi-based alloys, and the differences in their macro- and microstructures associated with the addition of Co suggest a different nature of fractures in the samples. Therefore, it is of particular interest to investigate the effect of cobalt on the fracture surface of porous samples.

**Figure 6:**
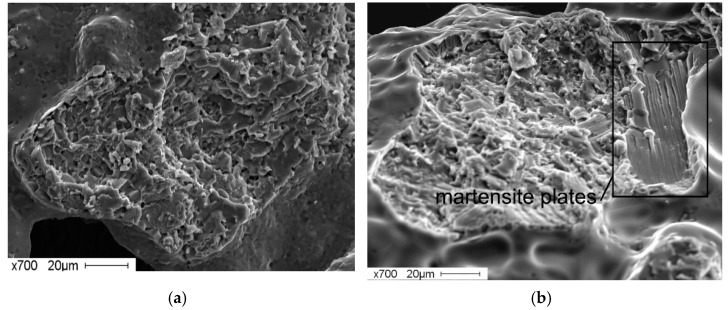
Fractograms of the fracture of porous TiNi-based alloy obtained by sintering without additives: (**a**) ductile-brittle nature of fracture; (**b**) mixed ductile-brittle nature of fracture with traces of B19′ martensite plates.

The surfaces of the fracture of a porous material without the addition of an alloying element are characterised by a set of areas of different fracture types within the sample volume (Figure 6a,b).

According to previous research [47,48,49,50], along with the B2 phase, the initial powder contains martensite phase B19′. Additionally, the investigation of the structural-phase composition of porous TiNi alloys, obtained by sintering in the present research, also shows the presence of austenitic and martensite phases (see Figure 2a). In some areas of the sintered material containing the B2-phase, a mixed ductile–brittle fracture surface topography is observed (Figure 6a). In other locations, a mixed-type topography of the fracture surface is also observed, consisting of cellular and lamellar martensitic topographies (Figure 6b).

**Figure 7:**
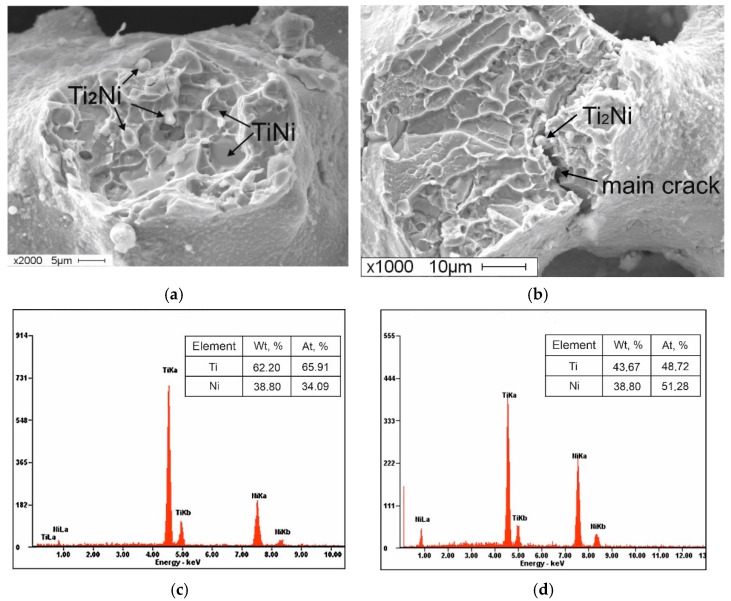
Fractograms of the fracture surface of the porous TiNi-based alloy with the addition of 0.5 at. % Co: (**a**) areas of intergranular fracture with traces of secondary Ti_2_Ni phases; (**b**) areas of fracture with traces of individual plates of B19′ martensite; (**c**) EDS of the secondary Ti_2_Ni phase; (**d**) EDS of the TiNi phase.

At 0.5 at. % Co, the mixed ductile–brittle fracture of the material is observed; this is characterised by the formation of intergranular fractures (Figure 7a). In the fractograms, one can observe the individual inclusions of particles of the phase that correspond to the elemental composition of the Ti_2_Ni phase (Figure 7a,c). At these inclusions, the main crack is likely to propagate (Figure 7b). In our opinion, the formation of an intergranular fracture is associated with the presence of accumulations of particles of Ti_2_Ni [51,52,53,54,55]. Furthermore, the system may contain individual fracture areas with traces of B19′ martensite plates.

**Figure 8:**
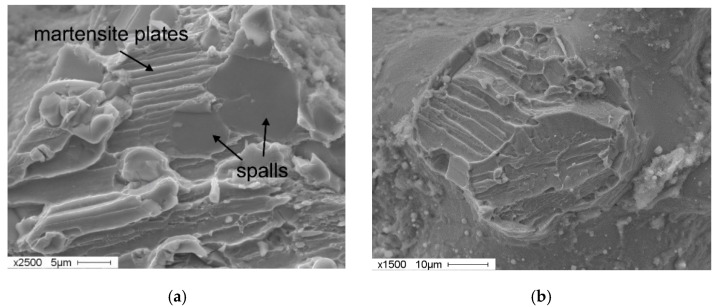
Fractograms of the fracture surface of the porous TiNi-based alloy with the addition of 1 at. % Co: (**a**) mixed fracture with the traces of a group of parallel plates and spalls; (**b**) change in the orientation of parallel plates in adjacent grains.

A peculiarity of the fracture of the sample with 1 at. % Co lies in the presence of extensive areas of a different type of mixed fracture in the structure. This type features traces of parallel martensite plates (corresponding to ductile fracture) as well as elements of spalls that are characteristic of brittle fracture (Figure 8a). Accumulations of elongated martensite plates have common orientations within one grain. The orientation changes when moving to an adjacent location (Figure 8b). The increase in the total number of areas with martensitic topography in the fractograms is associated with the increase in the proportion of the martensite phase, as the concentration of the additive reaches 1 at. %. Co in the initial state of the material before deformation (see Figure 2).

**Figure 9:**
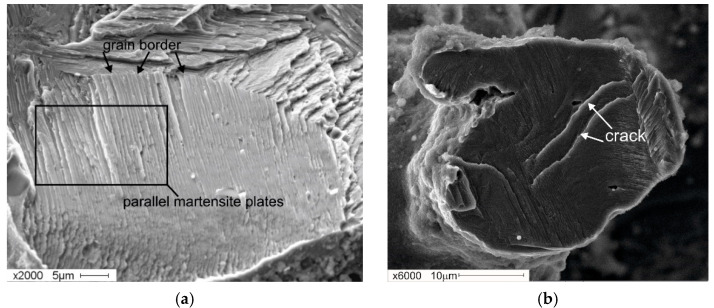
Fractograms of the fracture surface of porous TiNi-based alloys with the addition of 1.5–2 at. % Co: (**a**) intergranular spalling; (**b**) intragranular spalling and crack development along the intragranular (transcrystalline) plane.

It should be noted that, in porous samples with additive concentrations ranging between 1.5 and 2 at. % Co, almost the entire fractogram area is occupied by groups of parallel plates with a clear orientation that is limited by the grain. In this case, a fracture has either an intragranular nature or signs of intergranular spalling (Figure 9a). The formation of an intragranular spall is caused by the development of a crack along the intragranular (transcrystalline) plane, and this plane is normally represented by a specific crystallographic plane (Figure 9b). The extensive formation of martensite is facilitated by an array of finely dispersed strengthening Ti_3_Ni_4_ phases that create stresses in the austenite structure. According to the Clapeyron–Clausius equation, these stresses, caused by the deposition of finely dispersed coherent Ti_3_Ni_4_ particles, lead to the formation of B19′ martensite crystals [1,56].

It was shown that an increase in the number of regions, with the fracture of the martensite phase at 1.5 at. % Co, correlated with the extension of the deformation plateau, regarding the dependency σ(ε) of the given sample in the process of the merger of yield areas, σ1 and σ2 (see Figure 4). At the yield stage, martensite is formed. The process is initiated by deformation, i.e., strain-induced martensite is formed, which leads to the extension of this stage [57].

Thus, it was shown that the introduction of a cobalt additive leads to the evolution of fractograms of fracture of a porous TiNi-based alloy. From the data obtained, it was determined that an increase in the alloying additive in the range of 0.5–2 at. % Co changes the nature of fracture from a mixed, ductile–brittle fracture to a fracture with traces of martensite plates. At 2 at. %, these plates fill almost the entire fracture area.

## 4. Conclusions

In this research, the structural-phase composition of porous, TiNi-based alloys with cobalt additives obtained by sintering, was studied for the first time. The deformation behaviour of these materials was also studied for the first time with the use of tensile, and the fractograms of their fractures were analysed. Based on the data obtained, the following main conclusions were made:

1. It was determined that sintered, porous, TiNi-based alloys, with the addition of 0.5−2 at. % Co, have an almost identical structural-phase composition; the largest proportion of the martensite phase corresponds to 1.5 at. % Co concentration.
2. The main stages of deformation include sections of elastic deformations, yield points associated with martensitic transitions A → R and R → M, as well as areas of stress decays.
3. With the addition of 0.5 at. % Co, a sharp decrease in stress is observed, and this trend persists up to 1.5 at. % of the dopant. With an addition of 2 at. % Co, the stress rises to the level established for the samples with dopant concentration of 0.5 at. %.
4. It was determined that, on the one hand, the behaviour of the concentration dependency up to 1.5 at. % Co was associated with an increase in the stress in the TiNi solid solution, due to the arrangement of Co atoms on the Ti sublattice. On the other hand, it is associated with an increase in the fraction of the B19′ phase in the matrix phase.
5. It was found that a sharp increase in the martensitic shear stress at 2 at. % Co is attributed to the predominance of the precipitation hardening of austenite and an increase in the proportion of the austenite phase, following a decrease in the proportion of martensite.

The results of the effect of cobalt on the deformation behaviour and critical stress of the martensitic shear of a porous, TiNi-based alloy, can be used to achieve the required set of physical and mechanical properties for developing biocompatible implant materials.
